# Transverse Femoral Bone Transport for Refractory Diabetic Stump Ulceration: A Case Report

**DOI:** 10.2106/JBJS.CC.26.00235

**Published:** 2026-08-26

**Authors:** Alex Trompeter, Fabio Casero, Katie Hutchinson

**Affiliations:** 1 Trauma and Orthopaedics Department, St. George's University Hospitals NHS Foundation Trust, London, United Kingdom

**Keywords:** transverse bone transport, femoral amputation stump, diabetic ulcer, limb salvage, external fixation

## Abstract

**Case:**

A 40-year-old woman with diabetes presented with a nonhealing, ischemic through-knee amputation stump. To avoid transfemoral amputation, we adapted the transverse bone transport technique for the femur, modifying the surgical procedure by using a tibial fixator. A dual-cycle cortical distraction protocol successfully induced local angiogenesis, transforming the avascular tissue into a granulating wound bed and allowing for primary closure and limb salvage.

**Conclusion:**

Previously described only in the tibia, this first application of transverse bone transport provides compelling early evidence, 8 months postoperatively, that distraction-induced angiogenesis is a feasible and promising limb-salvage strategy for refractory amputation stumps at the femoral level.

Nonhealing wounds in diabetic patients present a major clinical challenge. Microvascular disease, neuropathy, and metabolic instability restrict tissue repair, often leading to chronic ulceration and proximal amputation^1^. Despite multidisciplinary management, treatment failure remains common with substantial functional and survival implications.^2^

Distraction osteogenesis techniques can modulate local biological activity in ischemic tissues. Transverse bone transport is used almost exclusively in the tibia, where controlled corticotomy distraction enhances perfusion and stimulates angiogenesis. Studies report improved healing in refractory diabetic ulcers, suggesting a role for mechanically induced vascular regeneration in limb salvage^2,3^. However, its use has been confined to the tibial cortex, and the feasibility of applying this technique to other long bones remains unexplored.

This report describes the first documented use of transverse bone transport in the femur of a chronic nonhealing diabetic amputation stump. This procedure uses a novel dual-cycle cortical elevation technique involving sequential mediolateral distraction and return transport to enhance local angiogenic response. This case illustrates that the principles underlying tibial transverse transport can be technically applied to the femur, offering early evidence that it may represent a feasible limb-salvage alternative to hip disarticulation.

The patient was informed that data concerning the case would be submitted for publication, and she provided consent.

## Case Presentation

A 40-year-old woman with poorly controlled type 1 diabetes and advanced end-organ complications presented with a chronic nonhealing left through-knee amputation stump. Since 2016, a recurrently infected diabetic toe ulcer necessitated sequential, increasingly proximal amputations. However, persistent infection, poor perfusion, and delayed healing repeatedly undermined recovery.

The most recent operation, a through-knee amputation performed 9 months before presentation, was followed by wound dehiscence and chronic infection with *Staphylococcus aureus* and *Pseudomonas aeruginosa*. Despite culture-directed antibiotics, debridement, and advanced wound care, the stump remained open and colonized. An MRI scan demonstrated no osteomyelitis, confirming the failure to heal was primarily attributable to severe microvascular compromise.

**Fig. 1:**
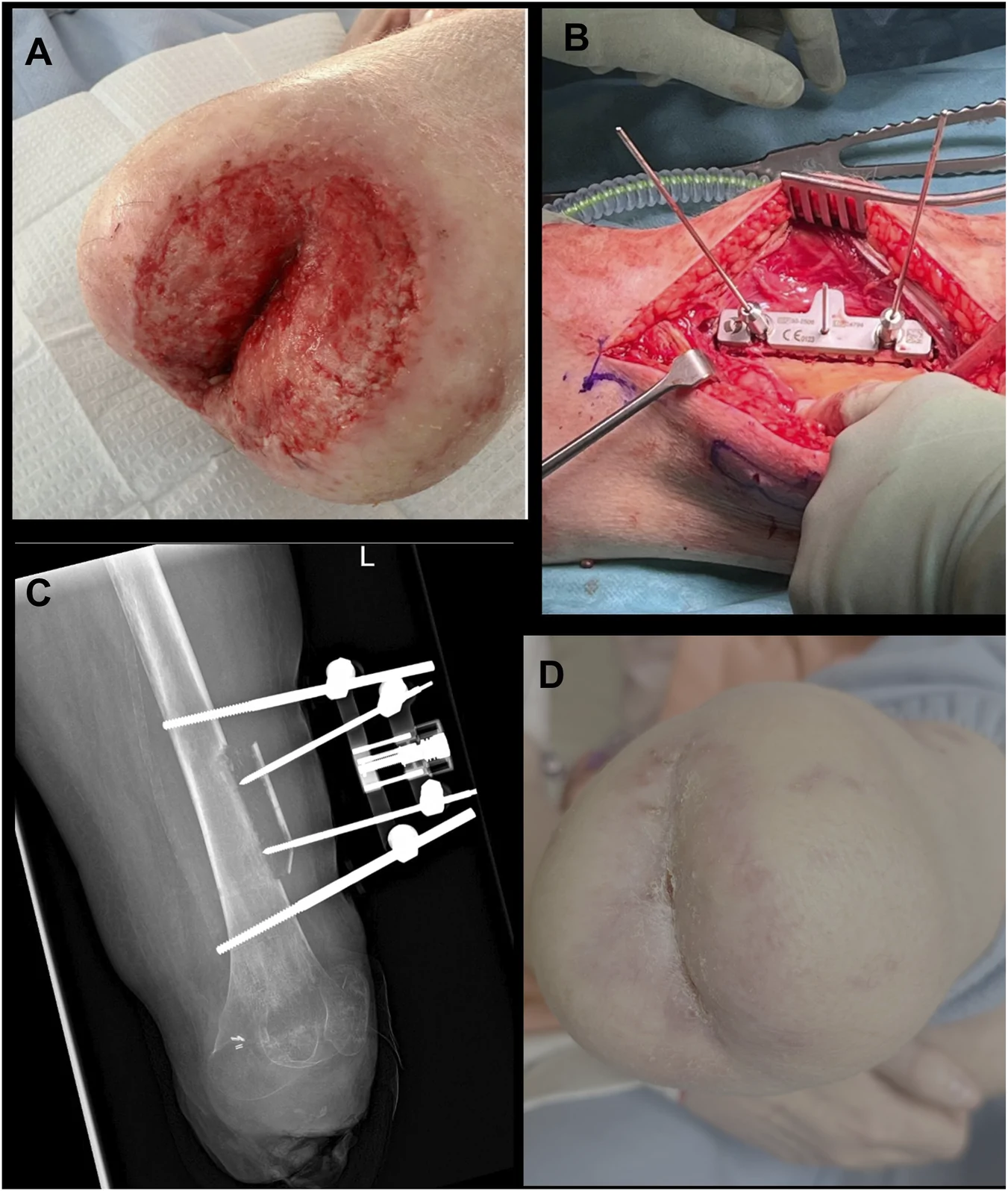
Application of transverse femoral bone transport for stump salvage. **Fig. 1-A** Preoperative view of the nonhealing, atrophic distal stump defect. **Fig. 1-B** Intraoperative view of the lateral femoral corticotomy and external fixator application. **Fig. 1-C** Radiograph during the distraction phase demonstrating elevation of the lateral cortex. **Fig. 1-D** Final clinical outcome showing a fully healed, stable stump (8 months postoperatively).

Over the ensuing 9 months, conventional strategies were implemented, including negative pressure wound therapy and glycemic optimization. Despite these efforts, the stump was deemed clinically refractory, defined by a total lack of epithelialization and failure to reduce in size (Fig. 1-A). A more proximal transfemoral amputation was deemed prohibitively high-risk; further shortening was unlikely to yield a healable stump and would severely compromise rehabilitation.^4^

### Surgical Management

After multidisciplinary review, we pursued a biologically targeted limb-salvage approach via distraction osteogenesis, adapting transverse bone transport for the femur.

An extended, curved lateral approach was used to mobilize the quadriceps envelope and expose the bone for corticotomy. A modified external fixation system (TrueLok Elevate, Orthofix) originally designed for tibial use was applied with minor technical adaptations (Fig. 1-B; see Supplemental Figure S1, http://links.lww.com/JBJSCC/C972). The osteotomy and the pins were all located on the anterolateral femur, just anterior to the iliotibial (IT) band. The IT band remained untouched, as its distal insertion was excised during the prior amputation and transverse transport does not induce longitudinal lengthening. Standard half-pins (4-mm elevation pins and 6-mm anchor pins) were inserted through separate stab incisions using guide wires.

After a 7-day latency, 2 complete transport cycles were performed over 2 months. Each cycle comprised 2 weeks of medial–lateral distraction followed by 2 weeks of reverse transport (Fig. 1-C). A second distraction-compression (“accordion”) cycle was initiated because, while the wound bed demonstrated robust granulation following the first cycle, it was not completely epithelized. This second cycle successfully drove the remaining tissue regeneration to completion (see Supplemental Figure S2, http://links.lww.com/JBJSCC/C972).

The stump showed progressive improvement, gradually filling with healthy vascularized granulation tissue (see Supplemental Figure S3, http://links.lww.com/JBJSCC/C972). Her medical (endocrine) management required an inpatient hospital stay; the surgical procedure did not influence this. Pin site care followed our standard protocol for all pin sites in our limb reconstruction unit.

The external fixator was removed 12 weeks postoperatively, with final primary closure about 4 months after the initial procedure. She was discharged to an amputee rehabilitation center 5 months after the initial surgery. At 8 months postoperatively, the patient maintains a well-healed, stable residual limb (Fig. 1-D) and has been successfully fitted for a prosthesis.

## Discussion

This case documents the first application of transverse bone transport in the femur for soft tissue reepithelialization in a diabetic patient. By transferring the principles of distraction-induced angiogenesis, this case demonstrates that biological mechanisms for tissue regeneration can be successfully reproduced in the proximal thigh.

Transverse bone transport is an effective biological therapy for refractory diabetic lower leg wounds^2^, correlating with increased ankle–brachial index and skin temperature. Complications remain infrequent.^3^

The rationale modifies Ilizarov’s distraction osteogenesis principle^5^. This movement generates powerful biological stimuli, upregulating angiogenic factors such as vascular endothelial growth factor and basic fibroblast growth factor, which significantly increase in serum postoperatively to promote neovascularization^6^. Extending the technique to the femur poses challenges due to thicker cortices and deeper soft tissue. However, our successful application suggests distraction osteogenesis effects are not site-specific.

Patients with refractory femoral stumps have limited options. While therapies such as vacuum-assisted closure can optimize ischemic stumps^7^, failures often culminate in hip disarticulation, carrying high perioperative risk and rendering patients wheelchair dependent^4^. By contrast, preserving femoral length through induced angiogenesis offers superior functional outcomes.

Given the successful biological healing demonstrated in this case, we suggest considering femoral transverse transport earlier in the management of complex stump wounds. Instead of reserving it strictly as a last resort, this proactive approach can salvage viable tissue before a patient requires a highly morbid procedure such as a hip disarticulation.

## Limitations

As a single case, these findings are hypothesis-generating. A key limitation is the absence of objective perfusion data. While clinical healing was observed, standardized vascular assessments were not performed to definitively substantiate the angiogenic mechanism. Furthermore, follow-up was limited to 8 months, and functional or prosthetic outcomes were not systematically assessed.

Future applications should incorporate objective hemodynamic measurements before and after distraction to confirm microcirculatory improvements, alongside long-term assessment of clinical and functional outcomes to define the precise role of femoral transverse transport.

## Learning Points

- Femoral transverse bone transport may be a feasible limb-salvage option for refractory soft-tissue defects in above-knee amputation stumps.
- Advanced imaging is essential to differentiate between deep-seated osteomyelitis and isolated microcirculatory compromise before attempting biological salvage.
- Standardized vascular assessments and functional outcome tracking should be incorporated in future applications to objectively confirm their mechanisms and clinical benefits.
- Transverse bone transport offers a potential alternative to high-level femoral amputation, with the aim of preserving the crucial femoral length.

## Appendix

Supporting material provided by the authors is posted with the online version of this article as a data supplement at jbjs.org (http://links.lww.com/JBJSCC/C972). This content was not copyedited or verified by JBJS.
